# Macular Hole Surgery with Internal Limiting Membrane Peeling Facilitated by Membrane-Blue® versus Membrane-Blue-Dual®: A Retrospective Comparative Study

**DOI:** 10.1155/2016/1292735

**Published:** 2016-12-05

**Authors:** Uri Soiberman, Daniel Shai, Anat Loewenstein, Adiel Barak

**Affiliations:** Department of Ophthalmology, Tel Aviv Medical Center and the Sackler Faculty of Medicine, Tel Aviv University, Tel Aviv, Israel

## Abstract

*Background*. This study aims to compare the outcome of macular hole (MH) surgery with internal limiting membrane (ILM) peeling facilitated by two different vital dyes.* Methods*. This was a retrospective chart review. The group designated “group-MB” underwent pars plana vitrectomy with ILM peeling facilitated by Membrane-Blue (MB), whereas in “group-MBD,” the vital dye used was Membrane-Blue-Dual (MBD).* Results*. Seventy-four eyes comprised the study population: 53 in group-MB and 21 in group-MBD. There was no difference in the rate of macular hole closure in group-MB or group-MBD: 71.2% closed MHs compared to 66.7%, respectively (*p* = 0.7). Postoperative visual improvement was of a higher magnitude in the MBD group compared to the MB group: −0.34 ± 0.81 logMAR versus 0.01 ± 0.06 logMAR, respectively (*p* = 0.003).* Conclusions*. In this study, MBD led to better visual results that may be related to better staining characteristics or lesser toxicity compared to MB.

## 1. Introduction

Macular hole surgery (MHS) is currently the mainstay for treatment of large full-thickness macular holes (FTMH) [1]. This procedure has evolved significantly since Kelly and Wendel initially reported a 58% anatomical closure rate [2]. Modern surgery includes pars plana vitrectomy (PPV) with or without internal limiting membrane (ILM) peeling and intraocular air/gas tamponade and leads to anatomical closure rates of 90% or more, especially when FTMH smaller than 400 *μ*m are treated [3]. The role of internal limiting membrane (ILM) peeling in the primary surgical treatment of this pathology is still controversial, but a growing number of surgeons employ this technique during the primary intervention in order to maximize the closure rate of the FTMH [4].

ILM peeling is a technically challenging procedure: the membrane is translucent and has a relatively low visibility. Many vital dyes have been suggested to facilitate the visualization of the ILM, such as indocyanine-green (ICG), trypan blue, and brilliant blue G® (BBG) [5–7]. In the past few years, two additional vital dyes have been introduced in clinical practice: Membrane-Blue (MB) and Membrane-Blue-Dual (MBD). MB consists of trypan blue 0.15%. It is commonly used in vitrectomy surgery to stain epiretinal membranes (ERMs), as well as the ILM. Prior studies with trypan blue or MB demonstrated macular hole closure rates exceeding 85% and a mean visual acuity improvement of at least two Snellen lines [6, 8, 9]. Other comparative studies of trypan blue or MB mostly demonstrated that when compared to ICG, both vital dyes led to similar macular hole closure rates (at least 84%) [10–15]. As for the visual acuity improvement, the results were inconclusive, with some studies reporting a better outcome with trypan blue/MB.

MBD consists of a combination of trypan blue (0.15%), brilliant blue G (0.025%), and 4% polyethylene glycol (PEG). This dye stains the ILM as well as other membranes (ERMs and proliferative vitreoretinopathy membranes), and it does not require an air-fluid exchange prior to dye injection because of its heavier molecular weight. A previous study comparing MBD to ILM-Blue®, another vital dye containing PEG that does not necessitate air-fluid exchange, has shown that there was no difference in visual outcome in eyes undergoing macular surgery with ILM peeling using either one of the aforementioned dyes [16]. However, macular hole was not the most common indication for surgery in that study.

MB and MBD were not compared directly in previous studies. This study aims to compare the outcome of MHS with ILM removal using MB versus MBD.

## 2. Materials and Methods

This was a retrospective study that was approved by the institutional review board (IRB) and was in accordance with the Helsinki Declaration of 1975, as revised in 2000. Since this study was retrospective, informed consent was waived by the IRB committee. All cases were identified from the operating-room log book in a tertiary referral academic medical center in the period between January 2010 and April 2013. All consecutive eyes that underwent pars plana vitrectomy with MB- or MBD-assisted ILM peeling for idiopathic FTMH were included. All patients were 18 years or older at the time of surgery. This study did not include eyes with a history of uveitis, endophthalmitis, or retinal detachment, nor eyes with other vision limiting pathologies. Cases of idiopathic FTMH in pregnant women were also excluded.

All operations were performed by one of two senior vitreoretinal surgeons (AL, AB). All PPVs were performed using a 23G transconjunctival trocar system and included a surgical induction of posterior vitreous detachment, a near-complete vitrectomy, a complete tamponade with C_3_F_8_, and postoperative head-down positioning for one week. Two groups were included in this study: group-MB—Membrane-Blue (DORC International, The Netherlands); group-MBD—Membrane-Blue-Dual (DORC International, The Netherlands). Both groups underwent similar surgeries, as stated above; however the MB group underwent air-fluid exchange prior to dye injection.

Study parameters included demographic data, lens status, and intraocular pressure (IOP). Additional parameters included presence of systemic hypertension and laterality (right or left eye). Best-corrected visual acuity (BCVA) was transformed from the Snellen acuity scale to logMAR. No light perception was set at logMAR 2.9, light perception at 2.6, hand motion at 2.3, and counting fingers at 1.85 [17, 18].

Surgical data included the type of vital dye used and noted cases in which PPV was combined with phacoemulsification and intraocular lens implantation. Postoperative data included the duration of follow-up, final BCVA and improvement in BCVA, BCVA at 6 months following surgery (when available), postoperative lens status, and IOP. Complications were also documented, especially, glaucoma, retinal detachment, residual subretinal fluid, reoperation, posterior capsular opacification, choroidal neovascularization (CNV), and reinjection of gas.

Spectral-domain optical coherence tomography (SD-OCT) was performed with Heidelberg Spectralis OCT (Heidelberg Engineering, Heidelberg, Germany). All measurements were performed by one skilled ophthalmologist (US). First available preoperative scans were compared with last available postoperative scans. Central macular thickness was calculated using the Heidelberg Spectralis module. The macular holes' transverse dimensions were assessed at the center of the holes: both basal diameter and the aperture diameter were measured. Other data included the attenuation of foveal retinal pigment epithelium (RPE), defined as focal or diffuse discontinuation of the sub- or perifoval RPE layer, focal retinal nerve fiber layer (RNFL) defects, defined as focal or diffuse breaks or absence of the inner retinal layers, intraretinal cysts, defined as intraretinal hyporeflective spaces, and misalignment in the retinal layers surrounding the location of the hole and closure of the macular hole. The latter was defined as misalignment of the hyper- or hyporeflective bands normally observed to be continuous when imaged with OCT.

**Figure 1:**
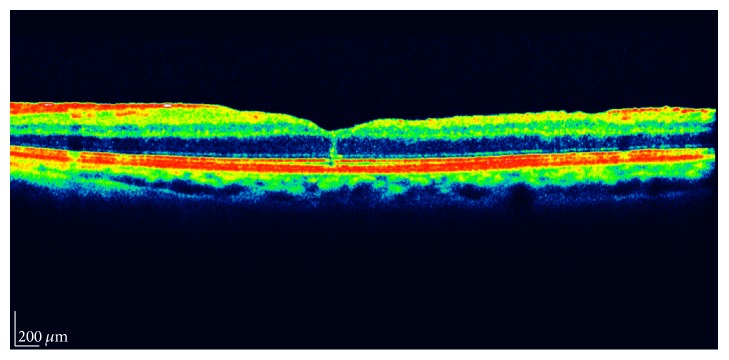
A vertical plane of separation is depicted anteriorly to the foveola in this spectral-domain OCT scan.

Postoperative scans were examined specifically for the existence of foveolar vertical planes of separation. A vertical plane of separation was defined as a hyperreflective axial line present in the foveola and extending from the retinal nerve fiber layer to Bruch's membrane (Figure 1).

*Statistical Analysis*. Statistical analysis was performed using SPSS software version 19.0 (SPSS Inc., Chicago, Illinois, USA). Continuous variables were examined for normal distribution by the Kolmogorov-Smirnov test, Q-Q plot, and histogram. Normally distributing parameters were described by mean and standard deviation, and the rest were described by median and range. A univariate analysis of the FTMH closure and categorical predictors was performed using Chi-square or Fisher's exact test. A *t*-test was performed for continuous, normally distributing parameters; the rest were tested with Mann–Whitney. Change in lens status between the two groups was assessed with McNemar's test. Visual acuity improvement analysis was performed with repeated measures ANOVA. A multivariate analysis for the outcomes was performed with a forward logistic regression with adjustment to universal confounders (age and gender with additional block). All tests were two-sided. A *p* value ≤ 0.05 was considered statistically significant; 0.05 < *p* ≤ 0.1 was considered a trend, due to the small size of the sample.

## 3. Results

Seventy-four eyes of 74 patients were included in this study: 53 in group-MB and 21 in group-MBD. Both groups had similar baseline characteristics, except for duration of the follow-up period, as illustrated in Table 1.

Information on the closure of the macular hole was available for 73 patients (data was missing for one patient in group-MB). Thirty-seven (71.2%) patients in group-MB had a closed macular hole by the end of follow-up compared with 14 (66.7%) in group-MBD (*p* = 0.7).

There was no baseline difference in BCVA between the MB and MBD groups (Table 1): 0.89 ± 0.54 logMAR (mean ± standard deviation; Snellen equivalent ~20/155) and 1.1 ± 0.67 logMAR (Snellen equivalent ~20/250, *p* = 0.17), respectively. When all study participants were included, BCVA improved from 0.95 ± 0.67 logMAR (Snellen: ~20/178) at baseline to 0.86 ± 0.55 logMAR (Snellen: ~20/145) at the last postoperative visit (*p* < 0.001). Visual improvement (the difference in mean postoperative BCVA at the last follow-up visit and preoperative BCVA) in the MBD group was greater than that of the MB group: −0.34 ± 0.81 logMAR versus 0.01 ± 0.06, respectively (*p* = 0.003). A similar trend was observed at 6 months postoperatively: −0.54 ± 0.63 logMAR for group-MBD (*n* = 17) versus 0.08 ± 0.33 logMAR (*n* = 45) for group-MB (*p* < 0.001).

Most of the study's participants did not undergo combined cataract extraction and vitrectomy—the procedure was only used on 4 eyes (all in group-MB). Nevertheless, at the end of follow-up there were 29 pseudophakic eyes in the MB group and 11 in the MBD group and a total of 24 eyes became pseudophakic during the follow-up period: 18 in the MB group and 6 in the MBD group (*p* = 0.6). The improvement in BCVA did not correlate with lens status exchange from phakic to pseudophakic during the study follow-up period (*p* = 0.26) nor with the final postoperative BCVA (*p* = 0.47).

The OCT findings differed between the two studied groups: a vertical plane of separation was evident in 19/53 (35.8%) eyes in group-MB and in 3/21 (14.3%) eyes in group-MBD (*p* = 0.07). Postoperative foveal RPE attenuation was detected in 34/53 (64.2%) eyes in group-MB and 9/21 (42.9%) eyes in group-MBD (*p* = 0.09). RPE attenuation was associated with the existence of systemic hypertension (OR 7.24, 95% CI 1.23–42.74, *p* = 0.03) and with laterality (right eye OR 0.12, 95% CI 0.02–0.56, *p* = 0.007). The compared groups did not vary in other studied parameters or complications (see Tables 2 and 3).

## 4. Discussion

The results of this study show that there was no difference in the rate of macular hole closure regardless of whether MB or MBD were used to facilitate ILM visualization during PPV and ILM peeling. Overall, there was a significant improvement in visual acuity, with the MBD group experiencing a higher magnitude of change. There was a trend for a vertical plane of separation in the MB group (*p* = 0.07), although the significance of the finding is unclear. A trend for postoperative foveal RPE attenuation was also detected in the MB group (*p* = 0.09). Associated risk factors were systemic hypertension and laterality, with the left eye being more affected (both surgeons in this study were right-handed).

The role of ILM peeling in macular hole surgery is still controversial. A recent meta-analysis failed to demonstrate a substantial benefit associated with ILM peeling: there was no difference in best-corrected distance visual acuity at 6 or 12 months postoperatively whether ILM peeling was performed or not [19]. The findings demonstrated that ILM peeling may be associated with primary hole closure after a single surgical intervention for macular hole stages 2–4 and also that fewer eyes with macular holes stages 2-3 necessitated repeat surgery if ILM peeling was performed. There was no difference between groups in the rate of intraoperative or postoperative complications. The findings suggest that ILM peeling should be reserved for eyes with prognostic factors for nonclosure. However, the meta-analysis did not specifically compare the outcomes for MBD versus MB.

If ILM peeling is to be performed, the optimal choice of vital dye remains in question. There is no data directly comparing all of the vital dyes commercially available, but many studies have compared some of them to ICG. A meta-analysis assessing the role of ICG in ILM peeling demonstrated that there was no significant difference between groups in anatomical outcomes (rate of primary, secondary, or final closure) whether or not ICG was used to stain the ILM [20]. However, visual acuity results were worse in the first 12 postoperative months in the ICG group. There was no difference in postoperative complications.

Another study comparing the results of MHS with ILM peeling using either BBG or ICG demonstrated that the mean BCVA and central (2°) retinal sensitivity were significantly better in the BBG group at 3 and 6 months postoperatively [21]. In the current study, eyes that had MBD staining had better visual results than eyes with MB staining. MBD contains BBG and TB; therefore the results support the findings of the previous report; however, the present study did not compare MBD to ICG.

A study comparing BBG, trypan blue, and ICG showed that, in eyes with macular holes stages 3-4, there was no statistically significant difference in anatomical closure rates [13]. At 6 months postoperatively, there were significantly more eyes in the combined BBG and TB group that had visual improvement in comparison to the ICG group. Also, visual acuity deterioration was significantly more common in the ICG group. That study reported that the participating surgeons described better ILM staining with BBG compared to TB, as well as easier ILM removal. Those results are also in compliance with the results of the current study, since MBD contains BBG and TB, and MB contains TB.

While the aforementioned studies suggest that ICG may lead to unfavorable surgical results, another study that compared BBG with ICG did not demonstrate a significant difference in postoperative BCVA between the two groups at 3 and 6 months [22]. Those results are contradictory to the current study's findings, where the MBD group had a better visual outcome.

A study comparing two heavy dyes, MBD and ILM-Blue, failed to demonstrate a difference in postoperative BCVA between the two groups at 1 month postoperatively; however, unlike the current study, macular holes were not the indication for surgery in the majority of the eyes included [16]. The lack of a difference between the two dyes may be the result of the incorporated PEG in both formulations. Our study compared a PEG-containing dye (MBD) to a non-PEG formulation (MB), which is the likely reason for the difference in visual outcome.

Dye toxicity becomes a substantial clinical consideration in ocular surgery, particularly when the toxicity surpasses efficacy. Trypan blue was previously reported to be toxic to the RPE: one case report suggested that reapplication of trypan blue caused progressive atrophy of the RPE in an eye that underwent MHS with ILM peeling [23]. Another case report associated trypan blue, light toxicity, or both with postoperative RPE atrophy [24]. Recent in vitro studies reported that trypan blue is nontoxic to the RPE [25, 26]. The present study demonstrated postoperative foveal RPE abnormalities that were more common (but not statistically significant) in the MB group. Considering the latest literature on trypan blue toxicity, these RPE abnormalities may be the product of increased dye concentration resulting from the air-fluid exchange, or they could even be the product of the air-fluid exchange itself.

This study has some limitations because of its retrospective nature. Additionally, the lack of substantial visual acuity improvement in the MB group and the low macular hole closure rate in both groups were disappointing. A recent study reported anatomical closure rates of approximately 90%; however all persistent macular holes were larger than 400 *μ*m [3]. That study failed to specify just how large the holes were. Another study assessing the risk factors for nonclosure following PPV and ILM peeling reported that the closure rate for Gass stage 4 idiopathic macular holes was 78.7%, and that those with a basal hole diameter of 800 *μ*m or above were 4 times more likely to persist [27]. The macular holes included in the current study had a wide range of basal diameter, as illustrated in Table 1, and some had very large diameters (>1000 *μ*m). Their mean basal diameters were 958.07 ± 347.1 *μ*m and 1012.93 ± 226.4 *μ*m for the MB and MBD groups, respectively; therefore the relatively low closure rates and lack of improvement in BCVA in the MB group may have been the result of selection bias.

In this retrospective study, eyes that underwent MHS with MBD-assisted ILM peeling had greater improvement in BCVA when compared with eyes that underwent MB-assisted ILM peeling. The difference between the two studied dyes may be attributed to higher contrast staining, which is usually associated with the use of MBD versus MB alone. This higher contrast may have resulted in more efficient ILM peeling, leading to better visual improvement; however there was no difference in hole closure rates between the two groups. The greater improvement in BCVA in the MBD group may also suggest that MB is more toxic to the retina than MBD.

## 5. Conclusions

In this study, MBD led to better visual results that may be related to better staining characteristics or lesser toxicity compared to MB.

## Figures and Tables

**Table 1:** Demographical and baseline characteristics.

Criteria	Membrane-Blue [*n*]	Membrane-Blue-Dual [*n*]	*p* value
Age (years)	66.83 ± 8.46 [53]	66.71 ± 5.62 [21]	0.95
Preop CMT (*µ*m)	405.08 ± 88.79 [40]	393.5 ± 72.39 [16]	0.64
Preop IOP (mmHg)	15.19 ± 2.45 [53]	14.1 ± 2.59 [21]	0.09
BCVA (logMAR)	0.89 ± 0.54 [53]	1.1 ± 0.67 [21]	0.17
MH aperture diameter (*µ*m)	403.82 ± 191.71 (range: 0–769) [44]	424.21 ± 128.08 (range: 230–637) [19]	0.62
MH base diameter (*µ*m)	958.07 ± 347.1 (range: 247–1981) [42]	1012.93 ± 226.4 (range: 739–1421) [15]	0.57
Duration of macular hole (months)	6.69 ± 7.34 [49]	5.4 ± 5.02 [20]	0.77
Duration of FU (days)	352.96 ± 364.66 (8–2327) [53]	95.05 ± 63.57 (9–211) [21]	<0.001

All data presented as mean ± standard deviation (range was also presented for duration of follow-up). Values within square brackets refer to the number of eyes with available data for analysis. CMT: central macular thickness; IOP: intraocular pressure; BCVA: best-corrected visual acuity; MH aperture diameter: the diameter at the narrowest point; MH base diameter: the base diameter of the macular hole; FU: follow-up.

**Table 2:** Postoperative parameters.

Criteria	Membrane-Blue	Membrane-Blue-Dual	*p* value
CMT (*µ*m)	287.4 ± 89.03 [45]	290.67 ± 59.34 [15]	0.18
IOP (mmHg)	14.45 ± 3.18 [52]	14.5 ± 3.14 [20]	0.9
Final BCVA (logMAR)	0.9 ± 0.56 [53]	0.76 ± 0.55 [21]	0.22
*In eyes with an open macular hole postoperatively:*			
MH aperture diameter (*µ*m)	582.5 ± 275.52 [10]	507 [1]	0.75
MH base diameter (*µ*m)	1136.3 ± 358.25 [10]	979 [1]	0.75

All data presented as mean ± standard deviation. Values within square brackets refer to the number of eyes with available data for analysis. CMT: central macular thickness; IOP: intraocular pressure; BCVA: best-corrected visual acuity; MH aperture diameter: the diameter at the narrowest point; MH base diameter: the base diameter of the macular hole.

**Table 3:** Postoperative OCT findings and complications.

Type of postop complication	Membrane-Blue *n* (%)	Membrane-Blue-Dual *n* (%)	*p* value
*OCT parameters*			
Vertical plane of separation	19 (35.8%)	3 (14.3%)	0.07
RPE attenuation	34 (64.2%)	9 (42.9%)	0.09
Misalignment of retinal layers	4 (7.5%)	1 (4.8%)	1
RNFL defects	34 (64.2%)	11 (52.4%)	0.35
Intraretinal cysts	12 (22.6%)	4 (19%)	1
*Clinical complications*			
Glaucoma	2 (3.8%)	1 (4.8%)	1
Retinal detachment	1 (1.9%)	0	1
Residual subretinal fluid	1 (1.9%)	1 (4.8%)	0.49
Reoperation	7 (13.2%)	1 (4.8%)	0.43
Posterior capsular opacity	1 (1.9%)	1 (4.8%)	0.49
Choroidal neovascularization	1 (1.9%)	0	1
Reinjection of gas	2 (3.8%)	0	1

OCT: optical coherence tomography; RPE: retinal pigment epithelium; RNFL: retinal nerve fiber layer.

## References

[1] Steel D. H. W., Lotery A. J. (2013). Idiopathic vitreomacular traction and macular hole: a comprehensive review of pathophysiology, diagnosis, and treatment. *Eye*.

[2] Kelly N. E., Wendel R. T. (1991). Vitreous surgery for idiopathic macular holes: results of a pilot study. *Archives of Ophthalmology*.

[3] Shah S. P., Manjunath V., Rogers A. H., Baumal C. R., Reichel E., Duker J. S. (2013). Optical coherence tomography-guided facedown positioning for macular hole surgery. *Retina*.

[4] Lois N., Burr J., Norrie J. (2011). Internal limiting membrane peeling versus no peeling for idiopathic full-thickness macular hole: a pragmatic randomized controlled trial. *Investigative Ophthalmology and Visual Science*.

[5] Burk S. E., Da Mata A. P., Snyder M. E., Rosa R. H., Foster R. E. (2000). Indocyanine green-assisted peeling of the retinal internal limiting membrane. *Ophthalmology*.

[6] Aguilera Teba F., Mohr A., Eckardt C. (2003). Trypan blue staining in vitreoretinal surgery. *Ophthalmology*.

[7] Enaida H., Hisatomi T., Hata Y. (2006). Brilliant blue G selectively stains the internal limiting membrane/brilliant blue G-assisted membrane peeling. *Retina*.

[8] Abdelkader E. A., McBain V. A., Anand M., Scott N. W., Siddiqui M. A. R., Lois N. (2011). In vivo safety of trypan blue use in vitreoretinal surgery. *Retina*.

[9] Li K., Wong D., Hiscott P., Stanga P., Groenewald C., McGalliard J. (2003). Trypan blue staining of internal limiting membrane and epiretinal membrane during vitrectomy: visual results and histopathological findings. *British Journal of Ophthalmology*.

[10] Schmid-Kubista K. E., Lamar P. D., Schenk A., Stolba U., Binder S. (2010). Comparison of macular function and visual fields after membrane blue or infracyanine green staining in vitreoretinal surgery. *Graefe's Archive for Clinical and Experimental Ophthalmology*.

[11] Christensen U. C., Krøyer K., Sander B. (2009). Value of internal limiting membrane peeling in surgery for idiopathic macular hole stage 2 and 3: a randomised clinical trial. *British Journal of Ophthalmology*.

[12] Beutel J., Dahmen G., Ziegler A., Hoerauf H. (2007). Internal limiting membrane peeling with indocyanine green or trypan blue in macular hole surgery: a randomized trial. *Archives of Ophthalmology*.

[13] Shukla D., Kalliath J., Neelakantan N., Naresh K. B., Ramasamy K. (2011). A comparison of brilliant blue g, trypan blue, and indocyanine green dyes to assist internal limiting membrane peeling during macular hole surgery. *Retina*.

[14] Bellerive C., Cinq-Mars B., Louis M. (2013). Retinal function assessment of trypan blue versus indocyanine green assisted internal limiting membrane peeling during macular hole surgery. *Canadian Journal of Ophthalmology*.

[15] Lee K. L., Dean S., Guest S. (2005). A comparison of outcomes after indocyanine green and trypan blue assisted internal limiting membrane peeling during macular hole surgery. *British Journal of Ophthalmology*.

[16] Veckeneer M., Mohr A., Alharthi E. (2014). Novel ‘heavy’ dyes for retinal membrane staining during macular surgery: multicenter clinical assessment. *Acta Ophthalmologica*.

[17] Holladay J. T. (1997). Proper method for calculating average visual acuity. *Journal of Refractive Surgery*.

[18] Schulze-Bonsel K., Feltgen N., Burau H., Hansen L., Bach M. (2006). Visual acuities ‘hand motion’ and ‘counting fingers’ can be quantified with the Freiburg Visual Acuity Test. *Investigative Ophthalmology and Visual Science*.

[19] Spiteri Cornish K., Lois N., Scott N. W. (2014). Vitrectomy with internal limiting membrane peeling versus No peeling for idiopathic full-thickness macular hole. *Ophthalmology*.

[20] Wu Y., Zhu W., Xu D. (2012). Indocyanine green-assisted internal limiting membrane peeling in macular hole surgery: a meta-analysis. *PLoS ONE*.

[21] Baba T., Hagiwara A., Sato E., Arai M., Oshitari T., Yamamoto S. (2012). Comparison of vitrectomy with brilliant blue G or indocyanine green on retinal microstructure and function of eyes with macular hole. *Ophthalmology*.

[22] Kadonosono K., Arakawa A., Inoue M. (2013). Internal limiting membrane contrast after staining with indocyanine green and brilliant blue G during macular surgery. *Retina*.

[23] Jain S., Kishore K., Sharma Y. (2013). Progressive atrophy of retinal pigment epithelium after trypan-blue-assisted ILM peeling for macular hole surgery. *Indian Journal of Ophthalmology*.

[24] Saeed M. U., Heimann H. (2009). Atrophy of the retinal pigment epithelium following vitrectomy with trypan blue. *International Ophthalmology*.

[25] Arndt C., Meunier I., Rebollo O., Martinenq C., Hamel C., Hattenbach L.-O. (2010). Electrophysiological retinal pigment epithelium changes observed with indocyanine green, trypan blue and triamcinolone. *Ophthalmic Research*.

[26] Costa E. F., Barros N. M. T., Coppini L. P. (2013). Effects of light exposure, pH, osmolarity, and solvent on the retinal pigment epithelial toxicity of vital dyes. *American Journal of Ophthalmology*.

[27] Brockmann T., Steger C., Weger M., Wedrich A., Haas A. (2013). Risk assessment of idiopathic macular holes undergoing vitrectomy with dye-assisted internal limiting membrane peeling. *Retina*.

